# Editorial: The gut microbiome's role in gastric cancer: mechanisms and therapies

**DOI:** 10.3389/fmicb.2026.1804029

**Published:** 2026-02-25

**Authors:** Duygu Ağagündüz, Silvia Giono-Cerezo, George Grant

**Affiliations:** 1 Department of Nutrition and Dietetics, Gazi University, Ankara, Türkiye; 2 School of Biological Sciences, National Polytechnic Institute (IPN), Mexico City, Mexico; 3 Independent Researcher, Aberdeen, United Kingdom

**Keywords:** antibacterial therapy, dysbiosis, gastric cancer, gastric microbiome, *H. pylori*, tumor microbiome

Gastric cancer is a major global health challenge and responsible for significant mortality worldwide (Park et al., 2025). There are, however, major continental and regional differences in its incidence. For example, rates in Eastern Asia are around 30/100,000 population, but tenfold lower (~3/100,000 population) in Northern Europe and the USA (Bautista et al., Marashi et al., 2024). As with many other alimentary tract malignancies and disorders, an individual's risk of gastric cancer is multifactorial, involving genetic, immune, lifestyle, environmental, dietary and microbial risk factors (Fernandez et al., 2025., He et al., 2025., Marashi et al., 2024., Cao et al., Han et al., Zhao et al., Zhili et al.).

The aims of this Research Topic were to identify gastric microbial profiles linked to increased risk of gastric cancer, to elucidate mechanisms by which the key microbes, in particular *Helicobacter Pylori* (*H. pylori*), and their virulence factors and bioactive metabolites trigger or modulate neoplasia, and to evaluate the therapeutic potential of exogenous microbes or microbial factors to limit or prevent gastric cancer.

Gastric colonization by *H. pylori* occurs in around 40 per cent of the world's population but the bacterium triggers gastric cancer in only 1 in 20 people. Adverse genetic, environmental, lifestyle, or dietary factors predispose the latter individuals to gastric cancer and provide the conditions in the stomach that allow *H. pylori* to initiate and promote gastric inflammation, epithelial disruption, DNA damage, and neoplasia (Bautista et al., Sundar et al., 2025).

*H. pylori* expresses an array of virulence factors, such as Cytotoxin-associated gene A (CagA), Vacuolating Toxin A (VacA), flagella, adhesins (BabA, SabA, OipA), and cag Pathogenicity Island (cagPAI) that facilitate colonization of the stomach, bacterial attachment to the gastric epithelium, suppression and circumvention of local immune defenses, initiation of persistent local inflammation and disruption of the epithelial layer through impaired cell turnover and functional development (Bautista et al., Huo et al., Huang et al., Lv et al., Zhu C. et al.). For example, CagA stabilizes Programmed Death-Ligand 1 (PD-L1), and thereby facilitates immune evasion. VacA induces apoptosis of epithelial cells. *H. pylori* induces production of Transforming Growth Factor β (TGF β) and activates associated receptors and molecular signaling pathways. Furthermore, it activates the NF-κB and IL-6/STAT3 pathways and alters the balance between Th17 and Treg cells. In combination, these actions impair the normal immune protection of the gastric mucosa (Bautista et al., Huo et al., Huang et al., Lv et al., Zhu C. et al.). As a result, *H. pylori* causes chronic inflammation, a severe loss of epithelial layer integrity, and progression to chronic gastric disorders, including severe ulceration and neoplasia (Bautista et al., Lv et al., Zhang et al., Zhu C. et al., Marashi et al., 2024).

Elimination of *H. pylori*, particularly from individuals at high risk of gastric cancer, has been a target for many years and many therapies. It is based on single or multiple combinations of antibiotics, and anti-bacterial factors, including probiotics, immunomodulators, anti-inflammatory or competitive bacterial or plant metabolites which have been shown effective in limiting the actions of *H. pylori* and facilitating its elimination from the stomach (Mu et al., Huang et al., Huo et al., Jiang et al., Zhang et al.). Despite these current successes, antibiotic resistance in *H. pylori* is an increasing problem (Schulz et al., 2025; Zhu H. et al.), highlighting the urgency of developing alternate strategies to eliminate this bacterium.

In many patients, clearance of *H. pylori* prevents development of gastric cancer or significantly slows its progression (Fu et al.). However, in other cases, elimination of *H. pylori* has only a limited impact (Wiklund et al., 2025; Bautista et al., Lv et al.). These discrepancies are likely linked to the stage to which cancer has advanced prior to exclusion of *H. pylori* from the stomach. Removal of *H. pylori* before cancer onset or during its early stages is likely to be more effective than elimination at later stages, when cancerous growth has become, to many intents, irreversible or self-perpetuating.

An increased risk of gastric cancer is linked to gastric and oral dysbiosis and a preponderance of potentially pro-inflammatory bacteria associated with the stomach epithelium, including the genera *Streptococcus, Fusobacterium, Prevotella, Veillonella* and the species *Fusobacterium nucleatum* and *Lactobacillus fermentum*, caused by the local niche-altering effects of *H. pylori* (Bautista et al., Huo et al., Lv et al., Song et al., 2026.) In addition, significant levels of the genera *Serinicoccus, Desulfomicrobium, Brachybacterium, Dietzia, Alishewanella, Kytococcus, Rheinheimera* and the species *Propionibacterium acnes, Klebsiella pneumoniae* and *Lactobacillus salivarius* are found within gastric tumors (Jin et al., Lv et al.).

It is still unclear whether these non-*Helicobacter pylori* bacteria can directly initiate cancerous changes in the stomach or act indirectly by boosting the disruptive and cancer-initiating effects of *H. pylori*, even after *H. pylori* has been eliminated from the stomach. These latter actions may occur through further induction of local inflammation that supports development of neoplasia or via production of carcinogenic factors that promote cellular proliferation, such as N-nitroso compounds, or other cell modulatory bacterial metabolites, such as acetaldehyde, and amino acid derivatives (Huo et al., Jin et al., Lv et al., Zhang et al.). To compound matters, there are indications that these bacteria may also modify or impair the action or efficacy of some chemotherapeutic drugs (Huo et al., Lv et al., Marashi et al., 2024). Either way, the present findings indicate that anti-bacteria treatments for patients with even early-stage gastric cancer must be directed at these additional possible neoplasia-inducing bacteria, as well as *H. pylori*, whether this be with antibiotics, inhibitors/modulators of bacterial function or metabolite production, or probiotics, such as *Bifidobacteria* or *Lactobacilli*. Alone or in combination these can facilitate restoration of a healthy gastric microbiome and aid in attenuating neoplasia (Bautista et al., Cao et al., Jin et al., Jiang et al., Marashi et al., 2024; Zhang et al., 2025).

The composition of the gastric and intratumoral bacteriomes is currently being evaluated for use in predictive models of gastric cancer risk and severity (Chen et al., Jin et al., Marashi et al., 2024). An issue with this approach is that the bacterial species associated with the neoplastic gastric epithelium or tumor are likely to change significantly with neoplasia duration and severity. In addition, this approach ignores the potential involvement of the mycobiome and/or virome, which can have significant cancer-promoting or exacerbating effects, even though they are minor components of the microbiome (Huang et al., 2023).

Probiotics such as *Lactobacillus* have inflammation- and neoplasia-ameliorative actions in gastric cancer patients, although elevated levels of *Lactobacillus* species can also be associated with the neoplastic gastric epithelium or tumor tissue (Bautista et al., Cao et al., Jin et al., Jiang et al., Zhang et al., 2025). These apparent differences in mode of action may be strain-dependent or linked to adverse metabolic changes in the bacterium caused by adaptation to a harsh local environment. Alternatively, since *Lactobacilli* are usually anti-inflammatory, it is also possible that their high levels in gastric cancer tissues reflect the gastric microbiome's attempt to suppress epithelial or tumor inflammation. This may also explain the lower cancer burden observed when gastric tumors harbor high levels of *Klebsiella* and *Achromobacter* (Jin et al., Lv et al.), as several bacteria from these genera produce potent anticancer metabolites.

In conclusion, *H. pylori* is the primary inducer of gastric cancer in high-risk individuals, but its neoplastic actions are aided, promoted, or modulated by other pro- or anti-inflammatory, pro- or anti-neoplastic bacteria in the constituent gastric microbiome. The properties and metabolite profiles of these non-*H. pylori* bacteria need to be evaluated as part of the development of strategies to ameliorate or inhibit gastric cancer.
